# Unpacking Engineering Practices for Curricular Assessment: Aligning Lab Practices and Assessment Items Using the 3D-LAP

**DOI:** 10.1021/acs.jchemed.4c01567

**Published:** 2025-07-23

**Authors:** Hunter McFall-Boegeman, Steven J. Petritis, Jacob Starkie, Cara E. Schwarz, Mengqi Zhang, Melanie M. Cooper, Elizabeth L. Day

**Affiliations:** † School of Natural Sciences, 5352 Northwest Missouri State University, Maryville, Missouri 64468, United States; ‡ Department of Chemistry, 3078 Michigan State University, East Lansing, Michigan 48824, United States; § Department of Chemistry & Biochemistry, 12337 The University of Texas at El Paso, El Paso, Texas 79986, United States

**Keywords:** Second-Year Undergraduate, Organic Chemistry, Laboratory Instruction, Problem Solving/Decision Making, Testing/Assessment, Learning Theories, Science and Engineering Practices

## Abstract

Laboratory learning has been the focus of significant creativity for educators. Engaging students in “real world” contexts or using their knowledge in laboratory exercises in a meaningful way requires that students are engaged in the practice of science. The role of decision-making around this practice of science, say for the analysis of the sustainability or social consequences of that science, can be structured as the application of engineering practices. To provide a coherent definition of how to elicit students’ use of knowledge, we turn to three-dimensional learning (3DL) and its previous applications in laboratory learning. Specifically, we extend the three-dimensional learning assessment protocol (3D-LAP) to reliably detect three distinct engineering practices: defining problems, evaluating solutions, and designing solutions. This analysis was then used to compare two second-year organic laboratory curricula: one verification or “cookbook” style and one project-based.

## Graphical abstract

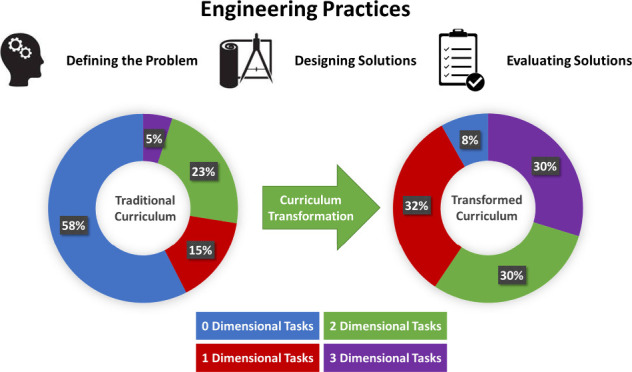

## Introduction

Preparing students to tackle global challenges, such as climate change or achieving the United Nations Sustainable Development Goals (UN SDGs), through the lens of chemistry has become a focus of many transformation efforts^2^ reported by several independent interventions.^3–8^ These efforts span introductory^9^ to upper division chemistry courses^10,11^ and include both laboratory^12,13^ and lecture^14^ courses. This focus on green and sustainable chemistry is often paired with environmental and sustainability issues or socioeconomic factors, using Systems Thinking or various other learning theories.^2,4,9,11^ There is evidence that as students move across a curriculum, these inconsistencies in design (ex. course culture, assessment focus, etc.) from different learning theories can negatively impact the effectiveness of the interventions.^15^ As such, it is important to design potential interventions for coherence with larger curriculum design efforts.

Herein we report on the expansion of previous transformations of courses at a large midwestern research intensive university using the Three-Dimensional Learning Framework (3DL).^1,16–20^ In our design framework, we focus attention on the practices of engineering within the context of chemistry and we demonstrate an approach that emphasizes chemical principles to solve problems that impact socioeconomic and environmental systems.^21^ By building upon previous transformations and implementing a coherent learning theory across courses, our goal is to support and provide evidence of how students think about global challenges as part of a larger transformation of an Organic Chemistry Laboratory.^21,22^

### Laboratory Curriculum Reform

A focus on learning in the chemistry laboratory has been extensively reviewed by Hofstein and Lunetta^23,24^ and Lowery-Bretz et al.,^25^ and even more broadly in science education (National Research Council’s *America’s Lab Report*).^26^ Typically the chemistry-specific reviews have highlighted that chemistry education research has been focused on students’ experiences,^27–30^ attitudes,^31–33^ and psychomotor skills,^34–37^ often in the service of meaningful learning. More recently, work has focused on how students engage with scientific practices in the context of sensemaking about phenomena.^1^ This expands the focus of research on laboratory learning to include what students can do with their extant knowledge, beyond descriptions of instruments and syntheses that students are asked for as part of verification laboratories. As Lowery-Bretz notes, the evidence for the importance, in terms of what students demonstrate that they learn, of laboratory learning is still needed.^38^

### Three-Dimensional Learning

3DL is a learning design framework from the consensus report *A Framework for K-12 Science and Engineering Education* (referred to hereafter as the *Framework*). Although originally developed for primary and secondary education, it is increasingly being adopted in higher education.^39–42^ The *Framework* highlighted three components to effective science education shown in Table 1. These dimensions are (1) Core Ideas (CIs), which is what students should know,^43^ (2) Cross-Cutting Concepts (CCCs), which are lenses/tools in which to frame questions/problems or epistemic heuristics,^44^ and (3) Science and Engineering Practices (SEPs), which is how students/scientists/engineers use their knowledge.^1^

**Table 1:**
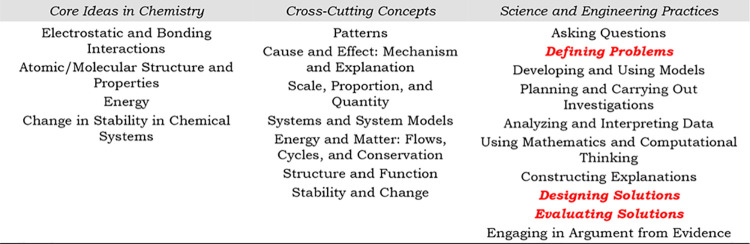
Core Ideas in Chemistry, Cross-Cutting Concepts, and Science and Engineering Practices from the Framework^47^ ^a^

a Engineering Practices expanded upon in this work are bolded, italicized, and red.

The *Framework* defined Scientific Practices (SPs) and Engineering Practices (EPs) similarly with the major difference being the framing of the question/problem students are addressing. SPs involve formulating a question that can be solved through scientific investigations (i.e., basic science research), while EPs involve formulating problems that can be solved through the engineering design process (i.e., applied research). Initial work on 3DL in higher education has focused on the Scientific Practices (SPs) of the SEPs.^45,46^ Despite this initial lack of incorporation, EPs are relevant to chemistry education as they can serve as a method of evaluating student decision making using their chemistry knowledge. This work seeks to expand on the EPs use in higher education.

Because of the context of an institutional transformational effort using a guiding design of 3DL across chemistry courses, we used the Three-Dimensional Learning Assessment Protocol (3D-LAP) as a metric of change. The 3D-LAP was developed for researchers (1) to characterize whether assessment tasks have the potential to elicit evidence of student engagement with the three dimensions and (2) to support faculty as they design assessment tasks.^46^ The 3D-LAP provides criteria a task must meet to be coded as capable of eliciting of CIs, CCCs, and SPs. However, because the original 3D-LAP was intended for use in lecture courses with summative assessment tasks, it did not include criteria for the EPs (defining problems and designing solutions) or communicating information; the creators of the 3D-LAP thought the practices were unlikely to be found on course exams.^46^ The EPs are relevant to laboratory activities and to contextualized instruction where students are asked to solve (real world) problems and to design solutions. Therefore, the primary focus of this article is to describe coding criteria for the EPs to extend the 3D-LAP. To demonstrate the utility of these coding criteria in identifying such practices, we use this extension to the 3D-LAP on the pre- and post-transformation curricula. This work was guided by following research aims.

## Research Aims

1. To develop coding criteria, from the defined learning objectives, for Engineering Practices that can be used to characterize the presence of EPs in laboratory activities and assessment tasks as part of the 3D-LAP
2. To explore how the style of laboratory activity and content focus affects the prevalence of different dimensions.

## Aim 1: Develop Criteria for Student Performance of Engineering Practices

### Methods: Unpacking of Engineering Practices and Developing Criteria for the 3D-LAP

In earlier work by Carmel et al., the authors recognized that omission of the EPs from the original 3D-LAP was problematic; this limited the tool’s applicability for activities in which students might reasonably be asked to design solutions to actual problems (rather than rote exercises), which lends itself well to laboratory tasks.^1^ In this work, they defined one EP by combining defining problems and designing solutions, which was identified by the criteria provided in the context of laboratory courses (see Box [Boxed-text box1-fo]). While this was an important first step in recognizing the importance of EPs in chemistry, combining defining problems and designing solutions into one practice meant that the criteria for this practice were overly broad.

1 **Previously Developed Criteria for Combined Engineering Practices^1^**

Students are asked to design/build something to serve a function as a result of their investigation

1. Activity gives an event, observation, phenomenon, scenario, or model.
2. Activity asks students to identify the findings from their investigation that will be used in designing the solution and how they will be used.
3. Activity asks students to design or build a tangible product from the results of their investigations
4. Activity asks students to discuss how they weighed or prioritized competing criteria, such as function, feasibility in production of design, and/or cost, in designing the solution

In our context, the use of EPs serves as a scaffold for making decisions about green and sustainable chemistry (GSC).^21^ We found it more useful to establish criteria that allow us to prompt for all the stages of the systematic practice of engineering design, that is (1) defining problems, (2) designing potential solutions and (3) evaluating potential solutions to problems akin to the Engineering Design core idea of the *Framework*.^22,47^ As such, our use of multiple EPs aligns with our design principles (Box [Boxed-text box2-fo]) of using GSC metrics and patterns of thinking to engage students in decision making about solutions to sustainability problems by allowing us to target each step of the Engineering Design process.^47^

2 **Design Principles for the Incorporation of Green and Sustainable Chemistry (GSC) into a Curriculum^21^**

Design Principle #1: The Underlying Chemical Principles of Sustainability Phenomena Should Be Emphasized and Supported

Design Principle #2: The Complexity of Sustainability Issues Addressed Should Be Increased over Time

Design Principle #3: Engagement with Engineering Practices Can Support Decision-Making

Design Principle #4: Focus Students’ Cognitive Efforts on the Important Ideas Rather than on Esoteric Tools and Metrics

We used a similar approach to the development of the 3D-LAP to develop criteria for the EPs: Defining Problems, Designing Solutions, and Evaluating (and optimizing) Solutions.^46^ Development began with a review of the relevant portions of the *Framework* ^47^ and the *Next Generation Science Standards* (*NGSS*).^48^ During the review, all authors participated in discussions to decide on what the EPs would look like from the perspective of student performance. From this, three of the authors (H.M.-B., S.J.P., J.S.) developed the coding criteria by determining what evidence would convince them that the task has the potential to elicit the practice, and the whole team concurred. The EPs, their student performances, and the coding criteria are summarized in Table 2.

**Table 2:** Consensus Definitions of the Three Engineering Practices and Their 3D-LAP Coding Criteria Developed from the *Framework* ^47^ and *NGSS*.^48^

Engineering Practice	Student Performance	3D-LAP Criteria for Constructed Response Assessment Tasks^a^
Defining Problems	Students identify real-world issues that can be addressed scientifically, specifying constraints, and specifying criteria for desired qualities of the solution	Task defines (or asks students to define) a design problem and any important considerations needed for an acceptable solution, including social, technical, and/or environmental concerns
		1. Task describes (or asks students to describe) the problem and the boundary conditions of the problem (e.g., scale, time point)
		2. Task gives (or asks students to give) context for why the problem matters
		3. Task defines (or asks students to define) the physical system and its components
		4. Task identifies (or asks students to identify) to whom this problem matters.
		5. Task specifies (or asks students to specify) what needs to be considered for an acceptable solution to the problems
Designing Solutions	Students generate a solution to real-world problems and provide evidence-based reasoning to support how the solution meets the needs of different stakeholders	Task proposes (or asks students to propose) a solution to the problem and provides (or asks students to provide) justification with data and scientific information
		1. Task restates (or asks students to restate) the problem
		2. Task specifies (or asks students to specify) what needs to be considered for an acceptable solution to the problem
		3. Task provides (or asks students to gather) all the relevant data and scientific information
		4. Task asks students to propose a solution based on the data analysis
		5. Task asks students to justify the solution with reasoning that uses data and scientific information
Evaluating Solutions	Students compare two or more potential solutions to real-world problems, decide which solution best meets the needs of the stakeholders and provide evidence to support their claim	Task uses (or asks students to use) data and scientific information to evaluate two or more possible solutions to the problem and decide on a solution
		1. Task provides (or asks students to generate) a list of what needs to be considered for an acceptable solution to the problem
		2. Task provides (or asks students to gather) all the relevant data and scientific information
		3. Task asks students to analyze the strengths and weaknesses of two or more possible solutions
		4. Task asks students to decide on a solution and provide reasoning for the solution

a Just as with the original 3D-LAP, separate criteria were developed for constructed and selected response items.^46^ Criteria for selected response assessment tasks are in Box SI1

### Defining Problems

Defining Problems emphasize that engineers solve real-world problems which must have artificial limits (constraints) that are a result of agreement between the stakeholders and the engineers. This student performance in Table 2 was adapted from the *NGSS* performance expectation: “HS-ETS1-1: Analyze complex real-world problems by specifying criteria and constraints for successful solutions.” (See Supporting Information for relevant excerpts).^48^ From this we identified five criteria that an assessment task should contain in order to engage students in this EP. Outlined in Table 2, an assessment task must ask students to describe: the constraints (criterion 1), what is necessary for a successful solution (criterion 5), and the system in which the problem exists (criterion 3). Additionally, criteria 2 and 4 prompt students to consider why this problem is important and who is affected, the groups who have a stake in deciding what constitutes a successful solution. As with the 3D-LAP, some of the criteria might be met as part of the expository “background” information included in the assessment tasks.

Figure 1 shows an assessment task from one of the case studies developed as part of the curriculum transformation^22^ and identifies the criteria for Defining Problems. In Figure 1, the assessment task prompts for the problem (different amide synthetic routes) to be defined from a sustainability perspective using the human and environmental hazards.^49^ First students to reflect on the boundary conditions (criterion 1) as they relate to the amide-forming synthetic routes, as well as the mechanistic account of the physical system (criterion 3). Students are asked to describe the problem (criterion 1) and its importance (criterion 2), particularly to stakeholders (criterion 4) whose needs shape requirements for an acceptable solution (criterion 5).

**Figure 1:**
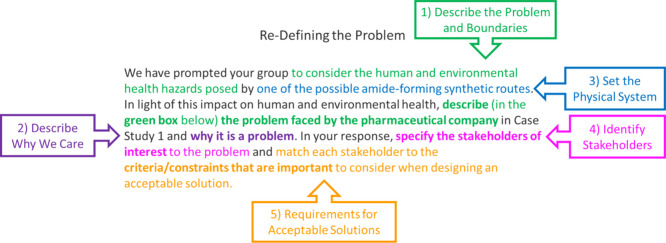
An example prompt from the new curriculum that was coded for the EP Defining Problems. Parts of the prompt satisfying the coding criteria are color coded to the corresponding criteria in callout boxes.

### Designing Solutions

Our student performance for designing solutions EP was adapted from the *NGSS* performance expectation: “HS-ETS1-2: Design a solution to a complex real-world problem, based on scientific knowledge, student-generated sources of evidence, prioritized criteria, and trade-off considerations.”^48^ The five criteria that a prompt must contain is shown in Table 2. The foundation of designing solutions is to understand the problem and the factors that affect it (criteria 1 and 2). Then, relevant data and scientific information can be collected (criterion 3) and the student proposes a solution (criterion 4) and justifies it based on the data collected (criterion 5).

Figure 2 shows an assessment task from a multiweek case study that satisfies the criteria for Designing Solutions. The task uses students’ responses to a previous assessment task, annotating a systems diagram, providing relevant data already collected (criterion 3). This example highlights two principles from our previously reported design framework (see Box [Boxed-text box2-fo]) in focusing students’ cognitive efforts (Design Principle #4) on engagement in the EP (Design Principle #3) by providing the relevant scientific information, as opposed to spending students’ time collecting that information themselves.^21^

**Figure 2:**
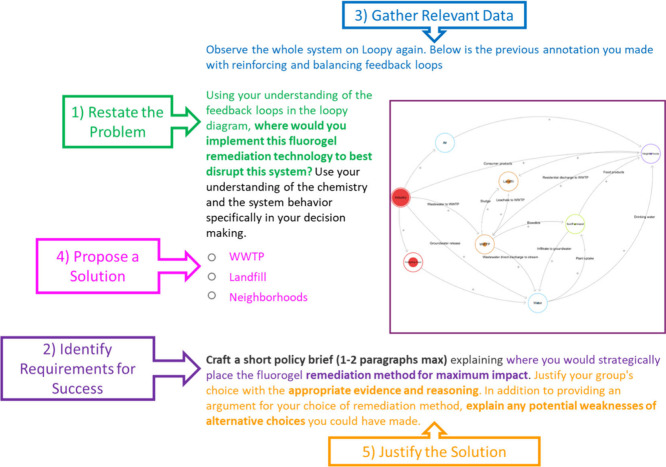
An example prompt from the new curriculum that was coded for the EP Designing Solutions. Parts of the prompt satisfying the coding criteria are color coded to the corresponding criteria in callout boxes. A higher definition version of the loopy diagram can be found in Figure SI1.

In the example represented by Figure 2, using the provided data, students must decide where to implement a remediation technology to disrupt the movement of fluorocarbons through the environment (criterion 1). Using the previous scaffolding and a system diagram, the students propose a solution by selecting a location in the system diagram to implement a remediation technology for maximum effect (criterion 4). Finally, the students write a short policy brief to justify their choice (criterion 5) by comparing their expected results with expected results of placing the remediation technology in different locations.

### Evaluating Solutions

Evaluating solutions is the final component of engineering design and is based on the premise that there is no single solution to a real-world problem as discussed in HS-ETS-3: “HS-ETS1-3: Evaluate a solution to a complex real-world problem, based on scientific knowledge, student-generated sources of evidence, prioritized criteria, and trade off considerations.”^48^ By evaluating different solutions, students must make decisions about which aspects of the problem are most important to the various stakeholders, and eventually come to a decision about which solution should be recommended. In our design framework, we have proposed supporting students using their knowledge to make evidence-based decisions is the richest performance of thinking about sustainability.^21^

Even if engaged in the Designing Solutions EP, Evaluating Solutions is a focus for analyzing how the other solutions could address the problem (Table 2). Based on the range of perspectives, various stakeholders (and the students themselves) may define the problem differently; this is incorporated into generating a list of what needs to be considered for an acceptable solution (criterion 1) and embeds significant decision-making, with conflicting needs from various stakeholders. To address how the proposed solutions meet the stakeholder needs and constraints, students should gather (or be provided) evidence about potential solutions to evaluate whether the proposed solutions meet the needs outlined (criterion 2). By comparing two or more potential solutions (criterion 3) students decide which solution best fits the problem definition from the perspective of stakeholders and communicate the chosen solution (criterion 4) to allow the stakeholders to review and validate the decision.

Figure 3 shows one of the scenarios of a project-based lab and how this artifact was analyzed for the coding criteria for Evaluating Solutions.^22^ In this scenario the students are asked to determine what the best source of caffeine for a marathon study session, given four sources of caffeine as potential solutions (criterion 3). Students must recommend one of the caffeine sources (criterion 4) by extracting caffeine (criterion 2) and comparing the amount of caffeine, its purity, and the price (criterion 1, identifying what needs to be considered for acceptable solution), using the cost-analysis tool.^50^ The stakeholder (the student) needs for a successful solution are explicitly defined for students as the maximum caffeine intake for the lowest price, which presents an opportunity for students to demonstrate decision-making through the practice of Evaluating Solutions.

**Figure 3:**
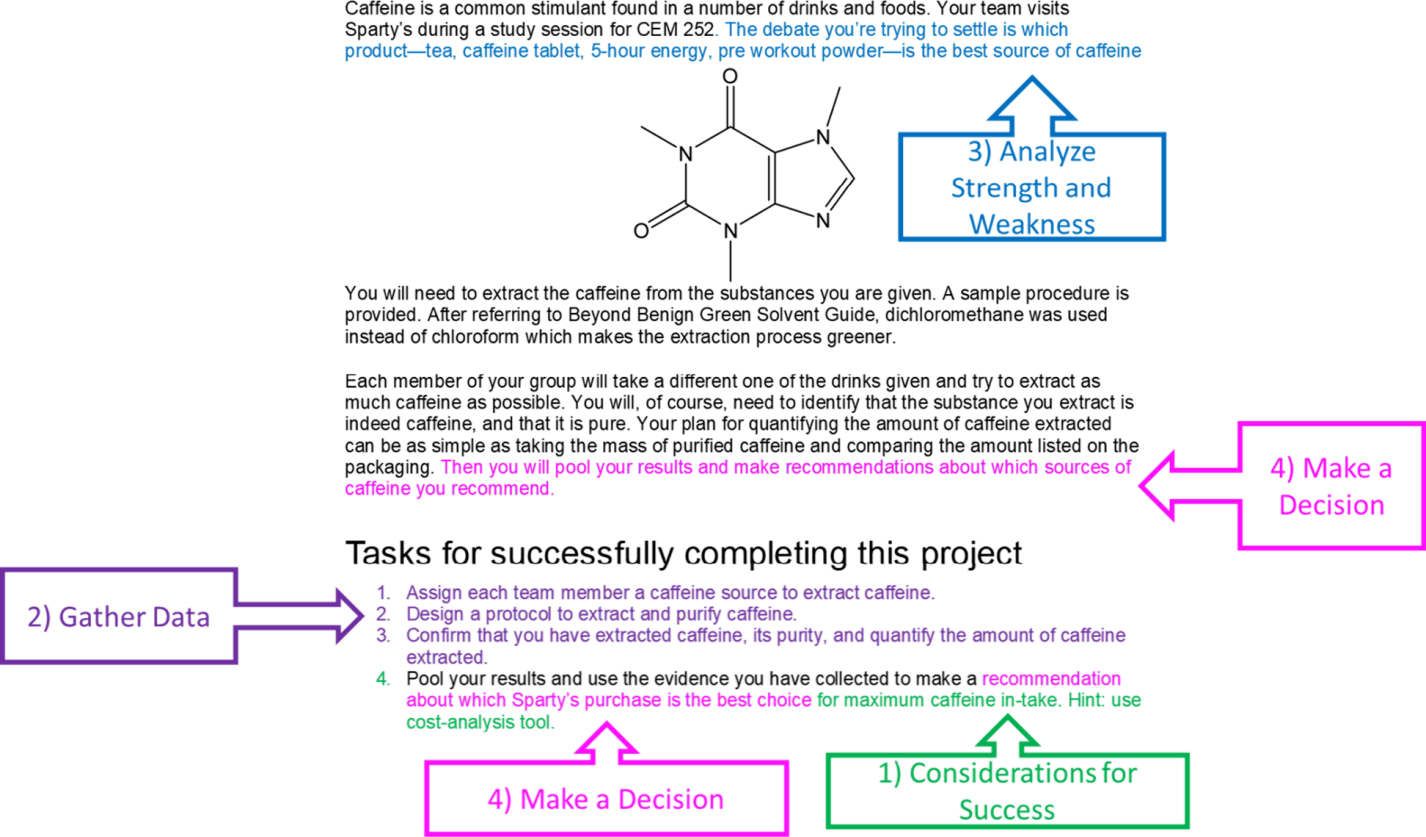
An example prompt from the new curriculum that was coded for the EP Evaluating Solutions Parts of the prompt satisfying the coding criteria are color coded to the corresponding coding criteria in callout boxes.

As shown in Table 2, the coding criteria for both Evaluating Solutions and Designing Solutions appear similar; the main difference between the two practices is the source of the solutions. Evaluating Solutions allows the instructor to customize the level at which students are interacting with the solution design and evaluation process to fit into their course context, to potentially lower the cognitive demand placed on students following Design Principle #4 (Box [Boxed-text box2-fo]) from our design framework.^21^ With this flexibility, potential solutions can be provided or students can generate their own potential solutions.

## Aim 2: Characterization of the Presence of Engineering Practices Using the Extended 3D-LAP

### Methods: Curricula Compared in this Study

To evaluate the utility of these EPs to extend the 3D-LAP, we determined the extent to which it can detect tasks that have the potential to elicit the three dimensions. We characterized the assessment tasks in two organic chemistry laboratory curricula, which were both implemented at the same large midwestern research intensive university and represented pre- and post- transformation time points as part of a larger curriculum transformation project.^21,22^ Titles and details on these activities are found in Table SI1 and Table SI2, respectively. This 2-credit course (approximately 600 students per semester) required students to attend a 1 h lecture in addition to 3 h in lab.

The pretransformation curriculum followed a traditional format of 13 weekly confirmatory investigations, using *CEM 255 Organic Chemistry Laboratory Manual*.^51^ In this curriculum, the lecture hour was used by the instructor to lecture to all the students in the course about that week’s experiment, and students were expected to read the lab manual, which outlined the techniques, theories behind the lab, and step-by-step procedures. The lab was assessed with several (1–7) short-answer questions as a worksheet.

The post-transformation curriculum is a version of *Cooperative Organic Chemistry*,^52,53^ a project-based lab which has an emphasis on GSC and additional case studies for the lecture hour. As previously reported,^22^ the laboratory projects are assessed through teams of students planning and executing an investigation to solve a given problem and write lab reports, and the case studies assess students in decision-making for a real-world problem in GSC.

### Coding Procedures

Initially, two authors (H.M.-B. and S.J.P.) coded both curricula (Table 3) for the presence of all the SEPs which includes the SPs defined in the original 3D-LAP protocol, the EP’s defined in Table 2, and the SP of communicating information as defined by Carmel et al.^1^ While the tasks that list laboratory procedures are unlikely to exhibit all three dimensions, we hypothesized that there could be SEPs such as “Designing and Carrying Out Experiments”, or “Analyzing and Interpreting Data” in the traditional verification-style laboratory curricula.^1^

**Table 3:**
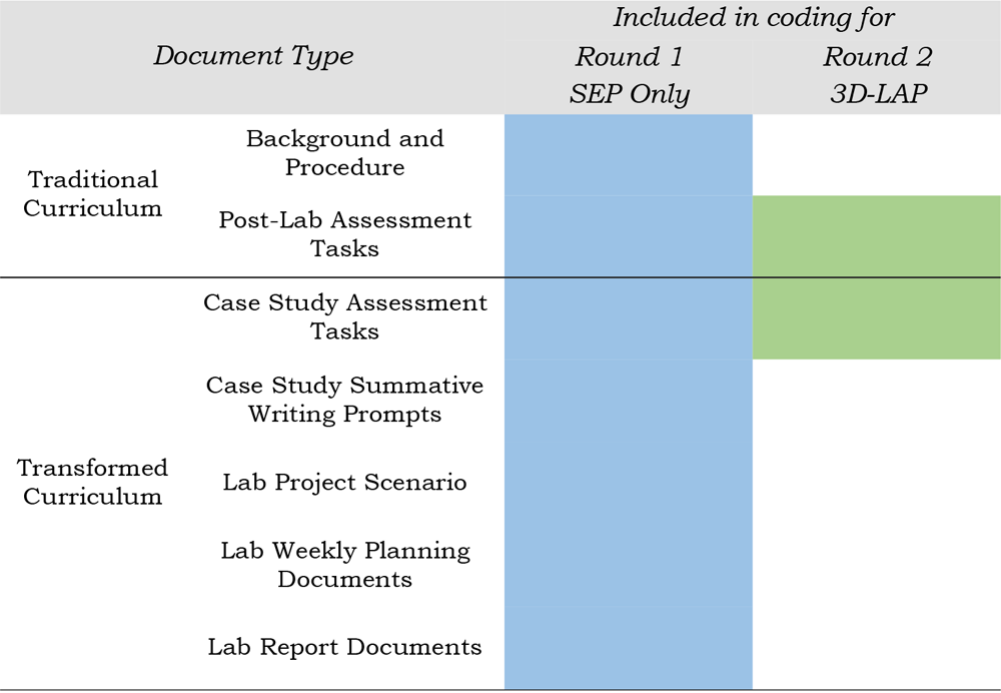
Types of Documents from Each Curriculum and Which Round(s) of Coding They Are Included In

The original 3D-LAP validation of coding criteria for SPs required only that coders agree that a scientific practice is present, not that coders agree that the same practice is present. In contrast, to validate the coding criteria developed for the EPs, both coders had to agree not only that a SEP was present but also agree which practice. To account for scaffolded prompts and clustered assessment tasks, all the assessment task prompts used per week in the curriculum were coded as a single unit.

Following the initial round of coding for the SEPs, the coders performed a second round of coding. In this second round of coding, the dimensionality of the case study assessment tasks for the transformed curriculum (37 assessment tasks over 3 case studies, Table SI2) and the postlab assessment tasks for the traditional curriculum (40 assessment tasks over 13 lab experiments, Table SIF) was determined (Table 3). These two document types were chosen because they were most similar between the two curricula, requiring students to answer constructed response questions most appropriate to be analyzed by the 3D-LAP.

Three authors (H.M.-B., S.J.P., and J.S.) independently coded tasks according to Table 3. Inter-rater agreement between the three coders was calculated using pairwise percent agreement in Excel prior to discussion of discrepancies. Inter-rater agreement was 84.8%, above the threshold established in the original report of the 3D-LAP.^46^ A consensus was reached through subsequent discussion of the discrepancies to reach 100% agreement, which did not alter any definitions from either the established 3D-LAP criteria or the coding criteria for new EPs. One author (C.E.S.) audited the consensus code from the 3D-LAP. Inter-rater agreement between the consensus code and the auditor was calculated using pairwise percent agreement in Excel. Inter-rater agreement was 82.3%, suggesting the original consensus code was valid.

## Results and Discussion

### Aim 1: To Develop Coding Criteria, from the Defined Learning Objectives, for Engineering Practices That Can Be Used to Characterize the Presence of EPs in Laboratory Activities and Assessment Tasks as Part of the 3D-LAP

Table 4 shows the results of the coding for SEPs for the traditional curriculum. As expected, most of the SEPs are absent, with the exception of Analyzing and Interpreting Data in experiments 3 and 10 and Communicating Information in experiment 12.

**Table 4:**
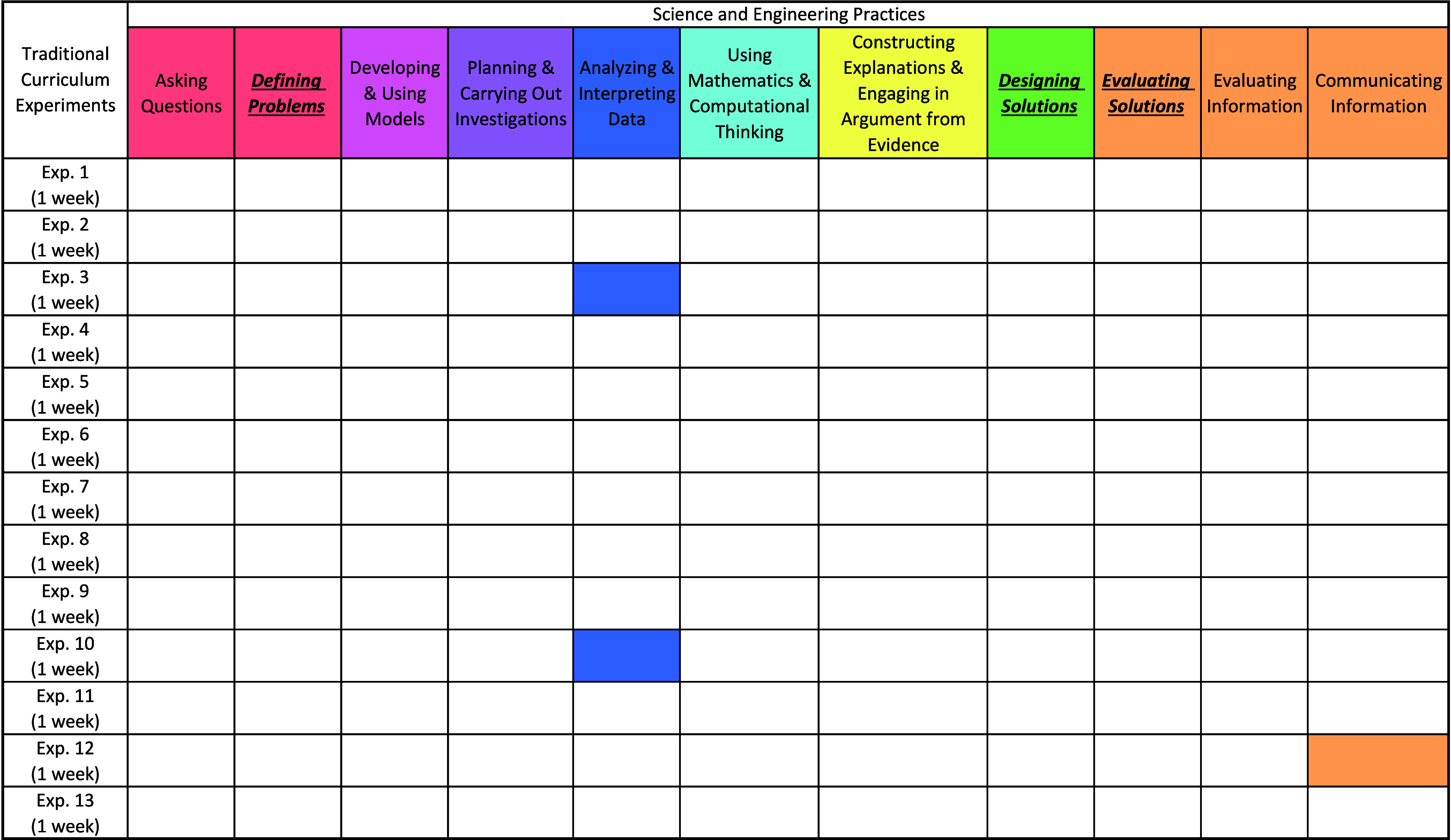
Results from Coding for All Science and Engineering Practices (SEPs) in the Traditional Curriculum Experiments^a^

a Highlighted practices indicate the experiment or case study has the potential to elicit the SEP. The newly defined Engineering Practices are **bolded**, *italicized*, and underlined.

In contrast, as shown in Table 5, every cluster of assessment tasks in the transformed curriculum had the potential to elicit at least one of the EPs. This curriculum was intentionally designed to engage students in SEPs, with an added emphasis on eliciting GSC decision making through the use of EPs. As such, we would expect these assessment tasks to elicit student engagement with SEPs in general and EPs in particular.

**Table 5:**
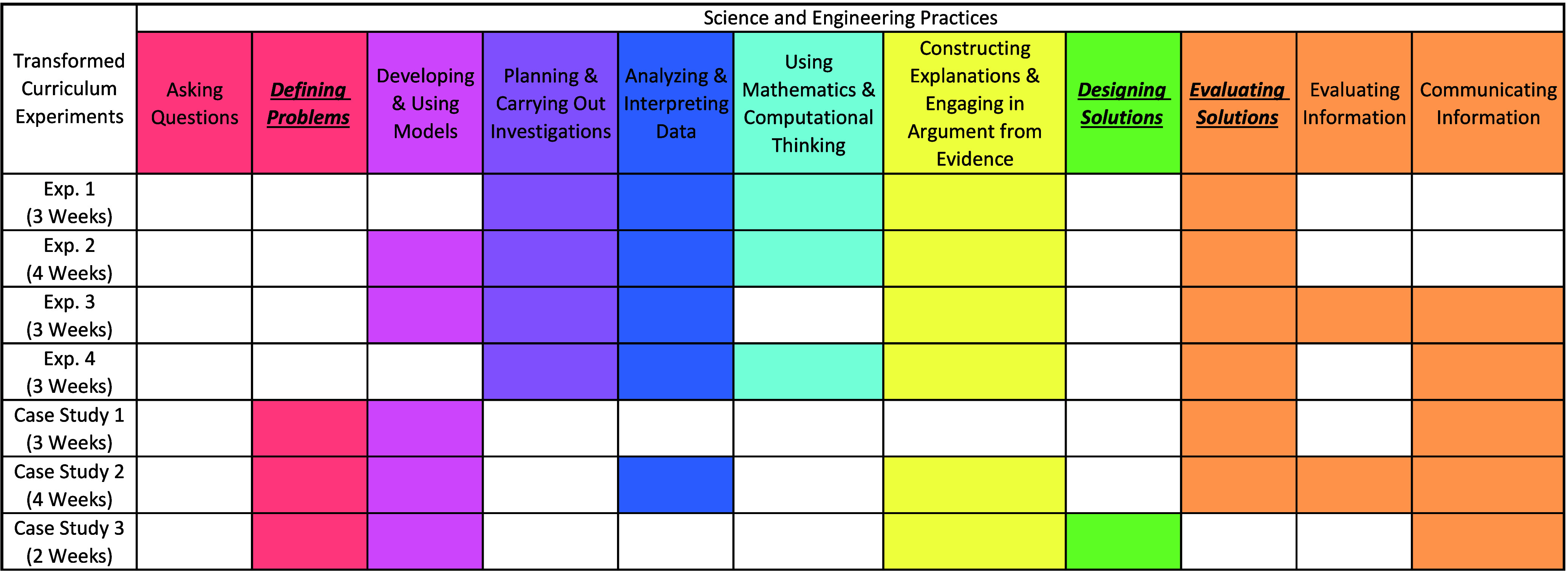
Results from Coding for All Science and Engineering Practices (SEPs) in the Transformed Curriculum Experiments and Case Studies^a^

a Highlighted practices indicate the experiment or case study has the potential to elicit the SEP. The newly defined Engineering Practices are **bolded**, *italicized*, and underlined.

### Aim 2: To Explore How the Style of Laboratory Activity and Content Focus Affects the Prevalence of Different Dimensions

Although the focus of this manuscript is not an in-depth analysis of the two curricula, two observations warrant discussion. The first observation is that engaging students in scientist and engineer-like behaviors needs to be deliberately addressed during the design process. As previously reported, the traditional tasks were mainly coded as 0- or 1-dimensional (Table SI4) and the SEPs were rarely present (Table SI5).^1^ Although one might imagine that laboratory activities can engage students in SEPs, in fact the traditional verification curriculum did not typically engage students in designing experiments or other SEPs.

The second observation is the differences in frequency of the dimensions in the transformed curriculum (Figure SI2 and Table 6), which was intentionally designed with 3DL. There is a different distribution of codes for assessment tasks capable of eliciting SEPs (26/37, 70.3%), CIs (19/37, 51.4%), or CCCs (22/37, 59.5%) compared to the traditional curriculum. Interestingly, in the transformed curriculum the CIs are least prevalent. This was unexpected based on previous uses of the 3D-LAP, where SEPs or CCCs are typically coded at a lower frequency.^46^ Because of this discrepancy we audited our results. Comparing the auditor’s codes with our consensus codes there was high pairwise percent agreement (82.3%) and that the least coded dimension was still CIs. We might ask why in the transformed curriculum CIs appear at a lower frequency.

**Table 6:**
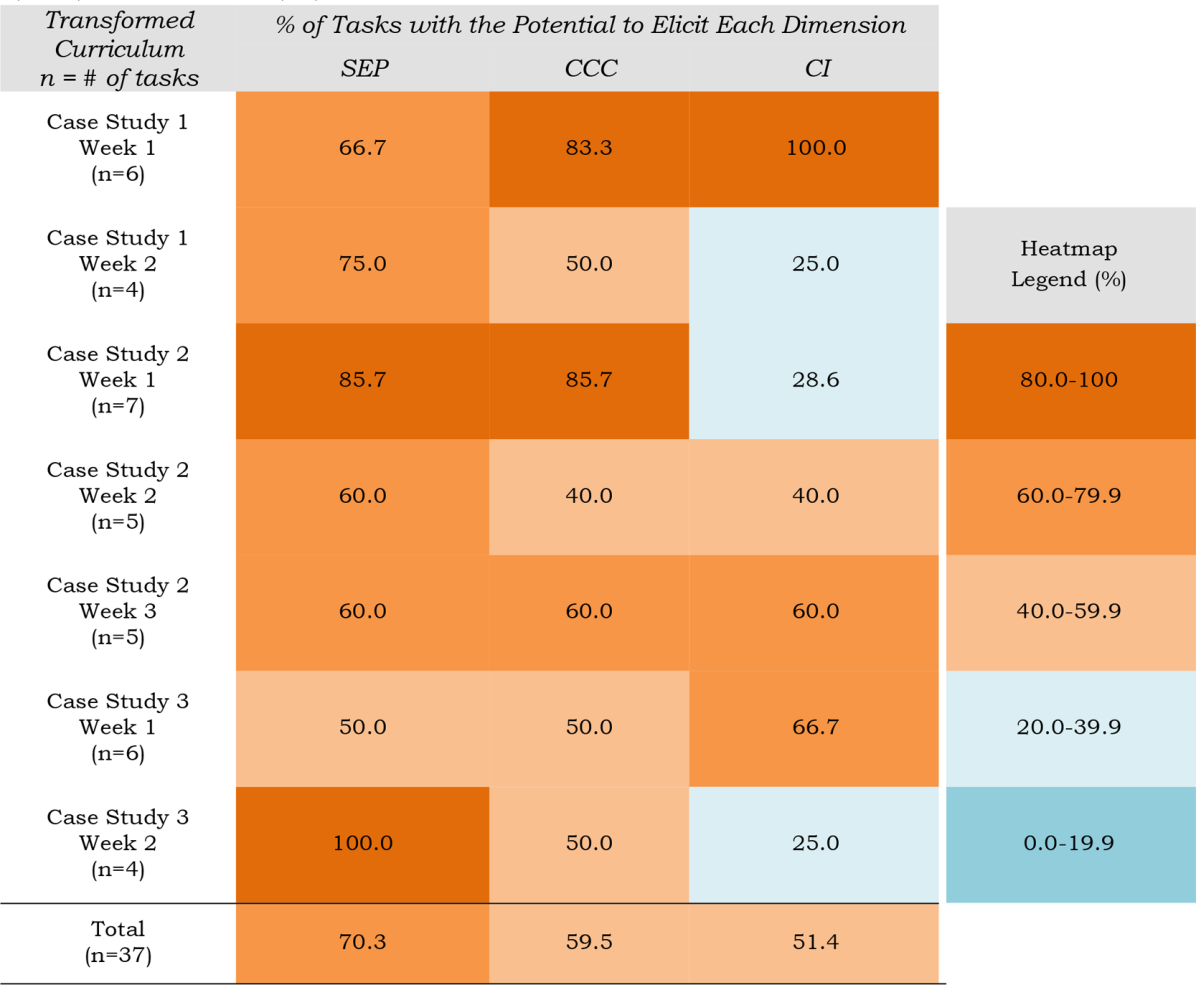
Percentage of Assessment Tasks in the Transformed Curriculum That Had the Potential to Elicit a Particular Dimension: Science and Engineering Practices (SEP), Cross-Cutting Concepts (CCC), or Core Ideas (CI)

To answer this question, we can look at the coding frequency for each dimension by week. Each case study is designed so students are first asked to develop a chemical model of the system (e.g., amide synthesis in case study 1, CS1). Then students consider the system from the perspective of different stakeholders and either design or evaluate potential solutions (i.e., evaluating different reaction routes in CS1).^21,22^ In two out of three case studies, week one has the highest frequency of codes for CIs compared to the other weeks (Table 6). For example, in CS1, 100% of assessment tasks in week one can elicit CIs whereas only 25% of assessment tasks in week 2 are potentially able to elicit CIs. Each week is scaffolded to support student decision making in the final student artifact, communication designed for a nonexpert audience (e.g., a white paper) which outlines a potential solution and their justification. As a result, the case study tasks clustered are capable of eliciting 3DL, even with fewer CIs as we move up the system scale. However, are tasks that lack engagement with chemistry CIs appropriate for a foundational chemistry course? In our view, to include GSC into the curriculum, it is important to design opportunities to engage with the SEPs (and the EPs in particular), but we must not neglect the chemistry underpinning those decisions (Design Principle #1, Box [Boxed-text box2-fo]).^54^

## Conclusions and Implications

In this paper we have used the *Framework* and the *NGSS* to help us design coding criteria for three engineering practices: Defining Problems, Designing Solutions, and Evaluating Solutions. These three practices can extend 3D-LAP so that it can be used to evaluate a broader range of instructional activities including laboratories and case studies.

We have used the expanded 3D-LAP to evaluate the potential of two different organic laboratory curricula to elicit 3DL and to look more closely at how the three dimensions are distributed. As expected, the traditional curriculum does not elicit 3DL, whereas the transformed curriculum more frequently engages students in the three dimensions. This finding aligns with previous results that traditional curricula are not designed using 3DL and do not elicit 3DL.^46^

Furthermore, for tasks that encompass GSC, we find that unless the activity is specifically designed to incorporate student engagement with the CIs of the discipline, tasks that engage students in the EPs are less likely to include chemistry content. This is an important point for curriculum designers: if we are to include EPs in the curriculum it will be necessary to intentionally design to emphasize the chemistry that underlies decision making.

## Limitations

One limitation is that we only evaluated two curricula. That limits the broader claims that we can make about the trends we observed related to GSC decision making and incorporation of CIs. It also means that we were not able to validate the EP coding criteria for selected response (multiple choice) assessment tasks. Neither the traditional nor the transformed curricula used selected response questions, but for many large enrollment courses, selected response tasks are common and convenient. As we hope to expand adoption of the curriculum, adopters are likely to adapt the material to their institutional context allowing access to a wider variety of tasks such as selected response tasks, which would allow the authors to validate the selected response criteria.

Additionally, although there is potential for a task to elicit SEPs, CCCs or CIs, it is not a guarantee that students will engage with the practices. The work reported above is not a substitute for scrutinizing the responses students produce.

Finally, as reconfirmed in our results above, a curriculum that is not implicitly designed to incorporate 3DL principles is unlikely to meaningfully incorporate the EPs. As previously reported, the 3D-LAP and the developed EPs may be less helpful for evaluating existing curricula as opposed to new curricula designed using 3DL principles as part of the iterative design process.^45,46^

## Supplementary Material



## Notes

Relevant excerpts from the *Framework*, coding criteria for engineering practices, raw data, and data analysis (PDF)
