# Supplementary material for: Unpacking Engineering Practices for Curricular Assessment: Aligning Lab Practices and Assessment Items Using the 3D-LAP
Source: J Chem Educ. 2025 Jul 23;102(8):3306–16. doi: 10.1021/acs.jchemed.4c01567 (PMC12355904; doi:10.1021/acs.jchemed.4c01567)
Supplement: Supplementary file 1 [file ed4c01567_si_001.pdf]

Supporting Information: Unpacking Engineering Practices for Curricular Assessment: Aligning Lab Practices and Assessment Items Using the 3D-LAP

Hunter McFall-Boegeman*[a], Steven J. Petritis[b], Jacob Starkie[b], Cara E. Schwarz[b], Mengqi Zhang[b], Melanie M. Cooper[b], Elizabeth L. Day[c]

[a]School of Natural Sciences, Northwest Missouri State University, Maryville, MO 64468, United States;

[b]Department of Chemistry, Michigan State University, East Lansing, MI 48824, United States;

[c]Department of Chemistry & Biochemistry, The University of Texas at El Paso, El Paso, TX 79986, United States

*E-mail: HUNTER@nwmissouri.edu

Building on our discussion of adapting NGSS[1] and the *Framework*[2] to generate 3D-LAP criteria, the following identify the relevant sections of the *Framework* to support the article text. For defining problems, our framing of the EP aligns with our goal of engaging students in sustainable decision-making, as demonstrated from the *Framework*:

> "Engineering begins with a problem, need, or desire that suggests an engineering problem that needs to be solved. A societal problem such as reducing the nation's dependence on fossil fuels may engender a variety of engineering problems, such as designing more efficient transportation systems, or alternative power generation devices such as improved solar cells. Engineers ask questions to define the engineering problem, determine criteria for a successful solution, and identify constraints."[2]

For the practice of designing solutions, we look to the *Framework* in terms of engineering design:

> "Engineering design, a systematic process for solving engineering problems, is based on scientific knowledge and models of the material world. Each proposed solution results from a process of balancing competing criteria of desired functions, technological feasibility, cost, safety, esthetics, and compliance with legal requirements. There is usually no single best solution but rather a range of solutions. Which one is the optimal choice depends on the criteria used for making evaluations"[2]

Box SI1. Criteria for Separated Engineering Practices

Defining Problems

Constructed Response

Task defines (or asks students to define) a design problem and any important considerations needed for an acceptable solution, including social, technical, and/or environmental concerns.

Ø Task describes (or asks students to describe) the problem and the boundary conditions of the problem (e.g., scale, time point, etc.).

Ø Task gives (or asks students to give) context for why the problem matters.

Ø Task defines (or asks students to define) the physical system and its components.

Ø Task identifies (or asks students to identify) to whom this problem matters.

Ø Task specifies (or asks students to specify) what needs to be considered for an acceptable solution to the problems

Selected Response

Task defines a design problem and asks students to select important considerations needed for an acceptable solution, including social, technical, and/or environmental concerns.

Ø Task describes the problem and the boundary conditions of the problem (e.g., scale, time point, etc.).

Ø Task asks students to select an appropriate response and reasoning for one of the areas below.

§ Select the context for why the problem matters.
§ Select the physical system and its components.
§ Select to whom this problem matters.
§ Select what needs to be considered for an acceptable solution to the problem.

Designing Solutions

Constructed Response	Selected Response
Task proposes (or asks students to propose) a solution to the problem and provides (or asks students to provide) justification with data and scientific information.<br><br>Ø Task restates (or asks students to restate) the problem.<br><br>Ø Task specifies (or asks students to specify) what needs to be considered for an acceptable solution to the problem.<br><br>Ø Task provides (or asks students to gather) all the relevant data and scientific information.<br><br>Ø Task asks students to propose a solution based on the data analysis.<br><br>Ø Task asks students to justify the solution with reasoning that uses data and scientific information.	We chose to remove Designing Solutions for the selected response tasks because, in our opinion, student selection of a preemptively designed solution and associated reasoning does not meaningfully demonstrate their engagement in this engineering practice.

Evaluating Solutions

Constructed Response	Selected Response
Task uses (or asks students to use) data and scientific information to evaluate two or more possible solutions to the problem and decide on a solution.<br><br>Ø Task provides (or asks students to generate) a list of what needs to be considered for an acceptable solution to the problem.<br><br>Ø Task provides (or asks students to gather) all the relevant data and scientific information.<br><br>Ø Task asks students to analyze the strengths and weaknesses of two or more possible solutions.<br><br>Ø Task asks students to decide on a solution and provide reasoning for the solution.	Task defines a design problem and provides data/scientific information needed to evaluate the given solutions to the problem. The student selects the "best" solution and either the data/scientific information that supports the decision or the appropriate reasoning.<br><br>Ø Task generates a list of what needs to be considered for an acceptable solution to the problem.<br><br>Ø Task provides all the relevant data and scientific information.<br><br>Ø Task assigns priority for the evaluation of possible solutions.<br><br>Ø Task asks students to select the "best" solution based on the data/scientific information provided.<br><br>Ø Task asks students to select the reasoning that links the appropriate evidence to their selected solution.

Table SI1. Traditional Curriculum Experiments		
Experiment #	Title	Description
Exp. 1	Paper Chromatography	Students use coffee filters to analyze dye colors in different inks via chromatography
Exp. 2	Melting Point	Students create a calibration curve for a Mel-Temp that will be used for the rest of the semester. Then identify an organic unknown by melting point determination
Exp. 3	Distillation of Ethanol/Acetone	Students compare simple and fractional distillation by separating a mixture of Ethanol and Acetone
Exp. 4	Acid-Base Extraction	Students separate 3-nitrobenzoic acid and methyl 3-nitrobenzoate based on pKa differences and recrystallize the separated compounds
Exp. 5	Preparation of Methyl 3-Nitrobezoate	Students synthesize and purify methyl 3-nitrobenzoate from methyl benzoate via electrophilic aromatic substitution

Exp. 6	Molecular Models Report	Students explore stereochemistry using physical 3D models
Exp. 7	Isolation of an Active Drug	Students isolate acetaminophen from tylenol via column chromatography and recrystallization
Exp. 8	Azo Dye Synthesis: Orange II	Students synthesize and purify the dye Orange II via diazotization
Exp. 9	Synthesis of Aspirin/Preparation of Soap	Students synthesize aspirin and soap via acetylation and saponification respectively
Exp. 10	Identification of an Unknown by Derivatization	Students identify an unknown aldehyde or ketone via melting point determination after derivatization with 2,4-dinitrophenylhydrazone and semicarbazide
Exp. 11	Structure and Reactivity of Carbohydrates	Students use 3D models and Benidict's solution to identify reducing and non-reducing sugars
Exp. 12	Consumer Product Project	Students create and present a poster describing an organic molecule found in a consumer product

Exp. 13	Grignard Reaction: Preparation of Triphenylmethanol	Students synthesize and purify triphenylmethanol by generating phenylmagnesium bromide in situ and reacting it with methyl benzoate

Table SI2. Transformed Curriculum Experiments & Case Studies		
Experiment/ Case Study #	Title	Description
Exp. 1	Organic Unknowns	Week 1: Plan and carry out an investigation to determine: (1) the solubility differences of the unknown compound in six greener organic solvents, and (2) the best solvent (mixture) for single solvent and binary solvent recrystallization of the unknown.<br>Week 2: Analyze and interpret qualitative and quantitative data to confirm the identity of the unknown.<br>Week 3: Write a Decision Memo to communicate the designed solution to the problem faced by the pharmaceutical company.
Exp. 2	Aspirin Synthesis	Week 1: Plan and carry out an investigation of the synthesis of aspirin using four different catalysts.<br>Week 2: Analyze spectroscopic data to confirm the identity of aspirin and use green metrics to design a solution for which synthetic route is highest yielding, the most economical, and affords the greenest synthesis.<br>Week 3: Analyze thin layer chromatographic data to determine the identity of binary analgesic mixture.

Exp. 3	Caffeine Extraction	Week 1: Design and execute a protocol for the extraction of caffeine from four different sources.<br>Week 2: Interpret data from three analytical techniques to confirm the identity and purity of caffeine. Generate an evidence-based argument for which caffeine source is most cost-effective.
Exp. 4	Amide Synthesis	Week 1: Design and execute a protocol for the synthesis of N-phenylacetamide using two different catalysts.<br>Week 2: Analyze spectroscopic data to confirm the identity of N-phenylacetamide and use green metrics to design a solution for which synthetic route is highest yielding, the most economical, and affords the greenest synthesis.
CS 1	Comparison of Synthetic Routes	Week 1: Develop a generalized model that explains amide bond formation.<br>Week 2: Define the problem faced by the pharmaceutical company and evaluate solutions from the perspectives of each stakeholder (Environmental, Accounting, and Chemistry teams).<br>Week 3: Write a Decision Memo to communicate the designed solution to the problem faced by the pharmaceutical company.

CS 2	Green-er Plastics	Week 1: Compare the monomer sourcing (beginning-of-life) of an oil-based and a plant-based polymer and construct a mechanistic explanation for their syntheses.<br>Week 2: Evaluate an argument that poly(lactic acid) (PLA) is compostable (end-of-life).<br>Week 3: Construct explanations and evaluate solutions for poly(ethylene terephthalate) (PET) recycling (end-of-life).<br>Week 4: Write a Policy Paper to communicate which area of Green-er Plastics research Congress should fund (beginning-of-life renewable sourcing vs. end-of-life recycling methods).
CS 3	PFAS	Week 1: Construct explanations of how the structure of PFAS molecules leads to both desirable and undesirable properties and define the emergent environmental problem.<br>Week 2: Define the PFAS problem in the broader (and more complex) local Michigan context and use a system diagram to design a solution for PFAS remediation.<br>Week 3: Write a Policy Brief to communicate the proposed remediation solution and reasoning for its implementation.

CS1 W1						
Slide #	5/6	7/8	10/11	13	15-17	18/19
SEP						
CC						
CI						

CS1 W2				
Slide #	2	5	6	8-14
SEP				
CC				
CI				

CS2 W1							
Slide #	9	11	12	14	15	16	17
SEP							
CC							
CI							

CS2 W2					
Slide #	5	6-8	9	10-12	13
SEP					
CC					
CI					

CS2 W3					
Slide #	5	8	9/10	11-13	14
SEP					
CC					
CI					

CS3 W1						
Slide #	4	6-8	10	11/12	15	16
SEP						
CC						
CI						

CS3 W2				
Slide #	6	7-9	12/13	14/15
SEP				
CC				
CI				

Figure SI1: 3D-LAP results of the transformed curriculum's Case Studies. Highlighted squares indicate a question can potentially elicit an SEP, CCC, or CI. Not all slides contained assessment tasks and were omitted from the tables. Similar to the original report of the 3D-LAP, question clusters were coded together and are noted.

To ensure that the new EP criteria did not affect the validity and reliability of the 3D-LAP protocol, the 3D-LAP coding results for both curricula were compared. The transformed curriculum was designed with the explicit intention to elicit EPs and just as we expected we found that many of the activities did incorporate SEPs (26/37, 70.3%, Table 6). In addition a number of them (11/37, 29.7%, Table SI3, Figure SI1) were shown to be 3-dimensional.[3] The traditional curriculum was not explicitly designed using 3DL principles, and as expected we found a lower prevalence of SEPs (4/40, 10.0%, Table SI5) and 3D tasks (2/40, 5.0%, table SI4) as shown in Figure SI2. The high-interrater reliability (84.8%) also suggests that addition of EPs to the 3D-LAP does not affect the reliability or validity of the 3D-LAP, when applied to our dataset. These findings, combined with results of the whole curriculum SEP coding, suggests that using the new EP criteria allows the user to detect tasks that are capable of eliciting the engineering practices of Defining Problems, Designing Solutions, and Evaluating Solutions.

Table SI3. The percentage of assessment tasks in the transformed curriculum that had the potential to elicit either 0, 1, 2, or 3-dimensions.

Transformed Curriculum	*% of Tasks with the Potential to Elicit that # of Dimensions*			
	0-Dimensions	*1-Dimension*	*2-Dimensions*	*3-Dimensions*
Case Study 1 Week 1 (n=6)	0.0	16.7	16.7	66.7
Case Study 1 Week 2 (n=4)	0.0	50.0	50.0	0.0
Case Study 2 Week 1 (n=7)	0.0	14.3	71.4	14.3
Case Study 2 Week 2 (n=5)	20.0	40.0	20.0	20.0
Case Study 2 Week 3 (n=5)	20.0	20.0	20.0	40.0
Case Study 3 Week 1 (n=6)	16.7	50.0	0.0	33.3
Case Study 3 Week 2 (n=4)	0.0	50.0	25.0	25.0
Total (n=37)	8.1	32.4	29.7	29.7

Heatmap Legend (%)
80.0-100
60.0-79.9
40.0-59.9
20.0-39.9
0.0-19.9

Traditional Exp. 1			
Q#	1	2	3
SEP			
CC			
CI			

Traditional Exp. 2			
Q#	1	2	3
SEP			
CC			
CI			

Traditional Exp. 3				
Q#	1	2	3	4
SEP				
CC				
CI				

Traditional Exp. 4			
Q#	1	2	3
SEP			
CC			
CI			

Traditional Exp. 5				
Q#	1	2	3	4
SEP				
CC				
CI				

Traditional Exp. 7	
Q#	1
SEP	
CC	
CI	

Traditional Exp. 8			
Q#	1	2	3
SEP			
CC			
CI			

Traditional Exp. 9			
Q#	1	2	3
SEP			
CC			
CI			

Traditional Exp. 10				
Q#	1	2	3	4
SEP				
CC				
CI				

Traditional Exp. 11		
Q#	1	2
SEP		
CC		
CI		

Traditional Exp. 12	
Q#	1
SEP	No Post Lab Questions
CC	
CI	

Traditional Exp. 13			
Q#	1	2	3
SEP			
CC			
CI			

Figure SI2: 3D-LAP results of the traditional curriculum's post-lab questions. Highlighted squares indicate a question can potentially elicit an SEP, CCC, or CI. Not all slides contained assessment tasks and were omitted from the tables. Similar to the original report of the 3D-LAP, question clusters were coded together and are noted.

Table SI4. The percentage of assessment tasks in the traditional curriculum that had the potential to elicit either 0, 1, 2, or 3-dimensions.

Traditional Curriculum	*% of Tasks with the Potential to Elicit that # of Dimensions*			
	0-Dimensions	*1-Dimension*	*2-Dimensions*	*3-Dimensions*
Exp 1 (n=3)	33.3	66.7	0.0	0.0
Exp 2 (n=3)	66.7	0.0	0.0	33.3
Exp 3 (n=4)	75.0	0.0	25.0	0.0
Exp 4 (n=3)	66.7	33.3	0.0	0.0
Exp 5 (n=4)	100.0	0.0	0.0	0.0
Exp 6 (n=7)	14.3	14.3	57.1	14.3
Exp 7 (n=1)	0.0	0.0	100.0	0.0
Exp 8 (n=3)	33.3	66.7	0.0	0.0
Exp 9 (n=3)	66.7	0.0	33.3	0.0
Exp 10 (n=4)	75.0	0.0	25.0	0.0
Exp 11 (n=2)	50.0	0.0	50.0	0.0
Exp 12	No Post Lab Questions			
Exp 13 (n=3)	100.0	0.0	0.0	0.0
Total (n=40)	57.5	15.0	22.5	5.0

Heatmap Legend (%)
80.0-100
60.0-79.9
40.0-59.9
20.0-39.9
0.0-19.9

Table SI5. The percentage of assessment tasks in the traditional curriculum that had the potential to elicit a particular dimension (Science and Engineering Practices (SEP), Cross-Cutting Concepts (CCC), or Core Ideas (CI)).

Traditional Curriculum	*% of Tasks with the Potential to Elicit Each Dimension*		
	SEP	*CCC*	*CI*
Exp 1 (n=3)	0.0	0.0	66.7
Exp 2 (n=3)	33.3	33.3	33.3
Exp 3 (n=4)	25.0	0.0	25.0
Exp 4 (n=3)	0.0	33.3	0.0
Exp 5 (n=4)	0.0	0.0	0.0
Exp 6 (n=7)	14.3	85.7	71.4
Exp 7 (n=1)	100.0	100.0	0.0
Exp 8 (n=3)	0.0	66.7	0.0
Exp 9 (n=3)	0.0	33.3	33.3
Exp 10 (n=4)	0.0	25.0	25.0
Exp 11 (n=2)	0.0	50.0	50.0
Exp 12	No Post Lab Questions		
Exp 13 (n=3)	0.0	0.0	0.0
Total (n=40)	5.0	35.0	30.0

Heatmap Legend (%)
80.0-100
60.0-79.9
40.0-59.9
20.0-39.9
0.0-19.9

(1) States, N. L. *Next Generation Science Standards: For States, By States*; The National Academies Press, 2013.

(2) Council, N. R. *A Framework for K-12 Science Education: Practices, Crosscutting Concepts, and Core Ideas*; National Academies Press, 2012.

(3) Zhang, M.; Day, E. L.; McFall-Boegeman, H.; Petritis, S. J.; Cooper, M. M. Incorporation of Green Chemistry into Undergraduate Organic Laboratory Using Cooperative Project-Based Experiments and Case Studies. *Green Chem. Lett. Rev*. **2023**, *16*, 2183781.
